# Retraction: Production of Potent Antimicrobial Compounds from *Streptomyces cyaneofuscatus* Associated with Fresh Water Sediment

**DOI:** 10.3389/fmicb.2018.01681

**Published:** 2018-07-10

**Authors:** Frontiers Editorial Office

The Journal and Chief Editors retract the 25 January 2017 article cited above. Concerns regarding the reliability of the data presented in this manuscript were brought to the attention of the journal in April 2018. Following an investigation by the Editorial Office and Chief Editors of the journal, it was found that appropriate controls had not been used for some of the experiments presented. The article is therefore being retracted from the journal due to concerns with the reliability of the data. The authors do not agree to the retraction, and the notice.

